# A scoping review of social determinants of health curricula in post-graduate medical education

**Published:** 2019-07-24

**Authors:** Kimberly Hunter, Ben Thomson

**Affiliations:** 1 Department of Family Medicine, Global Health and Vulnerable Populations, University of Toronto, Ontario, Canada; 2 Department of Medicine, International Health Professional Education Working Group, Queen’s University, Ontario, Canada

## Abstract

Social determinants of health are responsible for 50% of ill health. The Royal College of Physicians and Surgeons of Canada CanMEDS role of “physician advocate” requires physicians to attain competency in this particular domain, but physician trainees feel this is not well covered in their training programs. This study performed a scoping review of social determinants of health curricula that had been described, implemented and evaluated in post-graduate medical education. A search using MEDLINE(OvidSP) database, with search terms “residency,” ”curriculum,” and “social determinants” with no age, language, and publication date restrictions was done. Researchers identified a total of 12 studies, all from the United States, in internal medicine (n=4), pediatrics (n=4), family medicine (n=2), or multiple (n=2) residency programs. Most curricula (n=8, 67%), were longitudinal, and most contained both patient or community exposure (n=11, 92%) and/or classroom-based components (n=10, 83%). Most (78%) curricula improved participant related outcomes, including exam performance, awareness regarding personal practice, confidence, improved screening for social determinants of health and referral to support services. Program specific outcomes were frequently positive (50%) and included resident satisfaction and high course evaluation scores, high representation of resident and faculty from minority groups, applicability of training to underserviced populations, and improved engagement of marginalized community members. When evaluated, academic outcomes were always positive, and included acceptance of scholarly projects to national conferences, publication of research work, grants earned to support health projects, local or national awards for leadership and community engagement, and curriculum graduates later pursuing related Masters degrees and/or establishing medical practices in underserved areas. Only one study reported a patient-related outcome, with advice provided by health care providers considered by patients to be helpful. Researchers used these results to design recommendations for creation of a post-graduate curriculum to address social determinants of health were provided.

## Introduction

The social determinants of health (SDOH) are “the conditions in which people are born, grow, live, work and age, and the wider set of forces and systems shaping the conditions of daily life.”^1^ These determinants are broad and include income, education, housing, food security, employment and job security, social safety net and inclusion, gender, race, early childhood factors, access to health services, aboriginal status, and disability. In Canada, 50% of ill health can be attributed to SDOH,^2^ a greater proportion than biology, genetics, or the health care system. Thus, it is essential that health care providers know how to identify patients at risk of ill health due to SDOH, know strategies and resources to address health disparities, and then advocate for patient access to these resources.

The Royal College of Physicians and Surgeons of Canada developed the “CanMEDS” framework to guide medical education and physician practice.^3^ The CanMEDS role of “Health Advocate” includes the physician’s competence to identify patients’ SDOH. Resident physicians feel that while it is important to include Health Advocacy in medical curricula, it is not currently well covered, perhaps because it is deemphasized relative to other CanMEDS roles.^4,5^ Barriers to effectively delivering health advocacy education include the lack of published materials, lack of clarity regarding the physician’s role, unclear learning objectives, insufficient role modeling, and lack of a reliable method of assessment of competency.^6^ Given the marked impact SDOH have on patient health, the paucity of effective SDOH education impairs a physicians’ achievement of competence and inevitably leads to detrimental effects on patient outcomes.

A working group of program directors from Canadian family medicine post-graduate training programs met in 2011 to identify global health competencies for Canadian Family Medicine Training.^7^ Similarly, medical students at three Canadian medical schools established e-learning as an effective tool to enhance global health knowledge.^8^ However, there remains a paucity of SDOH curricula interventions that are implemented and evaluated at the post-graduate level.

The primary objective of this study was to identify studies in which the intervention of a post-graduate medical education curriculum focused on SDOH has been described, implemented, and evaluated in any patient population with disparities in health status or access. The secondary objective was to generate recommendations to design an effective SDOH curriculum within the CanMEDS framework.

## Methods

### Search strategy of scoping review and study selection criteria

A literature search confirmed that a review with the same objectives had not previously been published. A professional librarian (EU) ran a search in MEDLINE(OvidSP) for articles describing curriculum development centered around the topic of Social Determinants of Health, in post-graduate medical education (Appendix A). Search terms included “residency,” “curriculum,” “competency based education,” “interdisciplinary studies,” “problem-based learning,” “social determinants of health,” “health status disparities,” “social medicine,” “health services accessibility,” “health equity,” “socioeconomic factors,” “medical students,” “medical education,” “program development,” “program evaluation,” “evaluation studies,” and “validation studies.”

Due to the lack of studies from outside the United States in the first search, an “additional search” was performed. Search terms included “health advocate,” “CanMEDS,” “internship,” “residency,” “curriculum,” “competency-based education,” “interdisciplinary studies,” and “problem-based learning.”

There were no age group, language, or publication date restrictions for either search (Appendix A). Studies that were references in articles, but not found in the primary search, were hand searched and collected.

### Data synthesis and analysis

We included studies if a curriculum intervention to teach SDOH in a post-graduate medical education program was described and evaluated, with outcomes reported. We described studies by author, participants, post-graduate specialty program, and country, intervention (format, content and activities), method of evaluation, and outcomes. Studies were excluded if they did not describe and evaluate an intervention, and if outcomes were not reported.

Studies were summarized in Excel 16.16.7 for Mac. Meta-analysis of data was not possible because of the heterogeneous nature of available studies’ interventions and outcomes. All data analysis was performed in Excel 16.16.7 for Mac.

### Ethics approval

No direct patient contact was made for this study, and all accessed studies are a matter of public record. Therefore, there was no requirement to apply to Health Science Research Ethics Board for this study.

## Results

**Figure 1:**
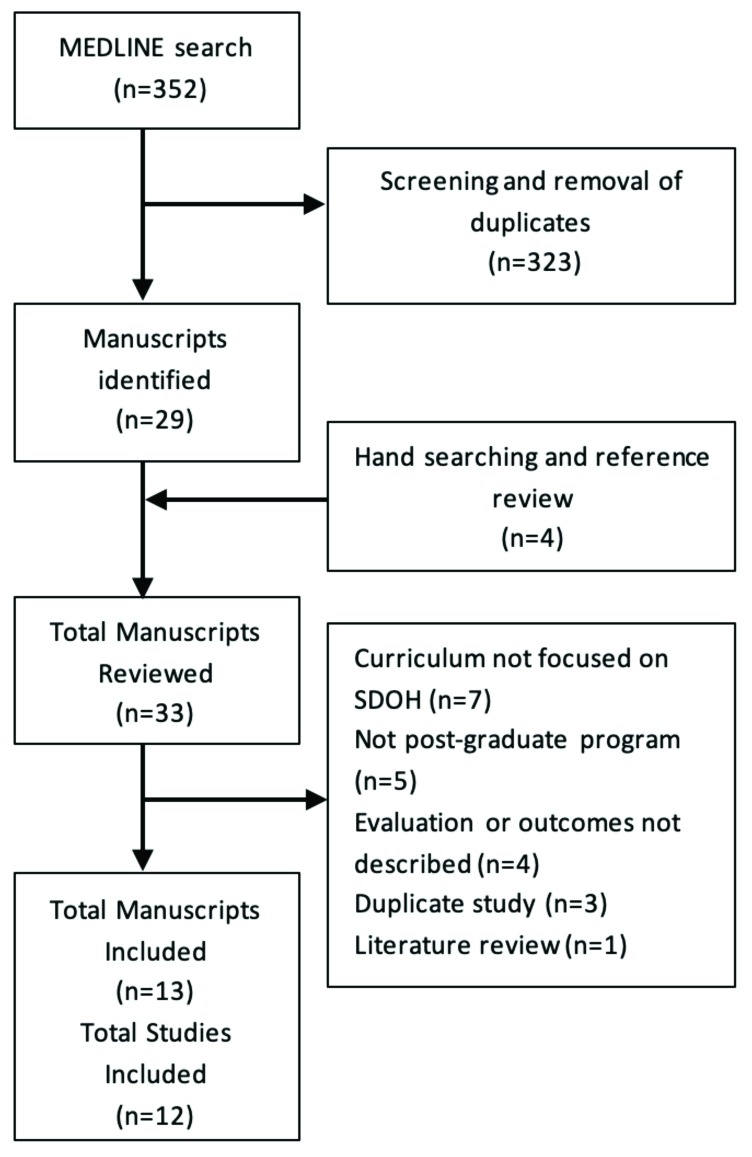
PRISMA flow chart for studies

Search of MEDLINE(OvidSP) yielded 352 references (Figure 1). Researchers screened references and removed duplicates yielding 29 manuscripts. Researchers added four references by hand searching and reviewing references. We reviewed 33 full manuscripts. There were 20 studies that were excluded, leading to 13 manuscripts describing 12 studies which were included in this review (Appendix B). Studies were excluded because the curriculum was not focused on SDOH (n=7), the study was not at the post-graduate level (n=5), the evaluations or outcomes were not described (n=4), the studies were duplicates (n=3) or a literature review (n=1).

An additional search yielded 333 references, leading to 15 manuscripts reviewed. None of these manuscripts met inclusion criteria, and all were excluded because they were not at the post-graduate level and/or did not describe a curriculum intervention and outcome.

### Participants post-graduate specialty program and country

Four studies (33.3%) were in pediatrics residency programs,^9-12^ while two (16.7%) were in family medicine,^13,14^ four (33.3%) were in internal medicine,^15-18^ and two (16.7%) were from multiple subspecialty residency programs (Appendix B).^19-21^ All studies (100%) were performed in the United States.

### Curriculum format and content

Eight studies’ curricula (67%) had longitudinal components that spanned across one or more year of residency (Table 1, Appendix B).^11,13-19,21^ These were organized either as blocks, incorporated either into ambulatory rotations or as separate tracks or specialties within a residency program. Three studies (25%) incorporated short interspersed learning components as modules, conferences, or half-day sessions.^10,12,20^ One study (8%) used a single-day learning experience.^9^ The curriculum content included resident needs assessment, literature appraisal, expert opinion and community input; however, this was too heterogeneous across studies to make any conclusions.

**Table 1:** Intervention characteristics

Characteristic	Frequency
**Format**	**n (%)**
Longitudinal	8 (67%)
Short Interspersed Learning	3 (25%)
Single Day Experience	1 (8%)
**Learning Activities**	
Patient or Community Exposure	11 (92%)
Classroom-based	10 (83%)
Independent Learning	5 (42%)
Project (research or advocacy)	5 (42%)
Other	4 (33%)

### Curriculum learning activities

Most studies’ curricula (92%) incorporated patient or community exposure through clinical practice, field trips, or patient interviews (Table 1, Appendix B).^9-11,13-21^ Most studies (83%) also included classroom-based activity which included didactic sessions, videos, resident-led teaching, small-group sessions, workshops, games, case-based simulation, role-playing, debates, or graduate-level courses.^9-12,15-21^ Five curricula (42%) required independent learning with literature appraisal, critical reflection, or assigned readings.^11,15,18-21^ Five studies (42%) had residents complete a research or advocacy project focused on vulnerable populations.^11,14-16,19,21^ Four studies (33%) used other forms of learning such as interprofessional collaboration or mentorship.^11,16,19^-^21^

### Evaluation methods

All curricula were evaluated using methods from one or more domains (Table 2, Appendix B). Most studies (75%) focused on participant outcomes such as frequency of SDOH screening, use of resources, referrals made to supporting services, knowledge assessments, measures of resident engagement, self-reflection, e-portfolios, achievement of core competencies, or pre/post surveys that assessed preparedness, skills, attitudes, competence, and knowledge.^9-12,14,16-19,21^ The majority (67%) of studies also evaluated program specific outcomes such as course evaluations, surveys of resident and faculty demographics, multisource feedback or analysis of the cost and time to implement the curricula.^11,13-15,17-21^ Academic benchmarks were evaluated in three (25%) studies, and included scholarly projects, publications, presentations at conferences, funding from donations or grants, and post-graduate career tracking.^15,16,19,21^ Patient-related outcomes were reported in only three (25%) studies, and included the results of patient surveys, the rate of outpatient clinic use and patient care outcomes (which were not further described).^10,12,14^

**Table 2:** Study evaluation type and outcome

Evaluation	Frequency n (%)	Positive outcomes n (%)
Participant-related	9 (75%)	7 (78%)
Program-specific	8 (67%)	4 (50%)
Academic benchmarks	3 (25%)	3 (100%)
Patient-related	3 (25%)	1 (33%)

### Evaluation outcomes

Studies that evaluated participant-related outcomes often (78%) reported positive outcomes (Table 2, Appendix B), which included improved competence or knowledge score on exams, increased awareness regarding personal practice, change in attitude or confidence, improved screening for SDOH with increased referral to support services, and increased exposure to mentors and potential career paths.^9-12,17-19,21^ Studies that evaluated program-specific outcomes reported positive outcomes in 50% of studies.^13-15,18^ These included high course evaluation ratings or resident satisfaction, high rates of representation of resident and faculty from minority groups, applicability of training and knowledge to care of underserviced populations, lack of need for additional funding required to implement curriculum, and improved engagement of marginalized community members agreeing to teach, to advise and to host field trips. All (100%) studies that evaluated academic benchmarks showed positive outcomes, with acceptance of scholarly projects to national conferences, publication of research work, grants earned to support health projects, local or national awards for leadership and community engagement, and curriculum graduates later pursuing related Masters degrees and/or establishing medical practices in underserved areas.^15,16,19,21^ Only one study (33%) reported a positive patient-related outcome, with advice provided by residents reported by patients to be helpful.^12^

## Discussion

In Canada, half of all ill health can be attributed to social determinants of health.^2^ Almost 15% of Canadians live at or below the poverty line, with marginalized populations disproportionately represented.^22,23^ Low income associates with shorter life expectancy, inability to afford prescribed medications, and higher incidence of chronic health issues such as diabetes and hypertension.^22,23^ However, physicians lack the training and resource knowledge to address these health disparities.^4-6^ This scoping review revealed 12 studies of curricula interventions focused on SDOH, with vastly heterogeneous program elements, evaluation methods and outcomes.

The most common curriculum format was longitudinal exposure throughout post-graduate training. This typically incorporated continuity of care clinics, encouraging familiarity and therapeutic alliance with the patient population and allied providers.^20^ These curricula developed comfort and knowledge of available resources, with improved understanding of the multiple aspects involved in the care of underserved populations.^13^ Curricula that were short and interspersed left trainees with improved understanding of the importance of SDOH, but the intervention was of inadequate duration to gain full understanding and competence on how to address these issues.^20^

Studies had varied outcomes and evaluation methods, with most focusing on participant or program specific elements. Most of the emphasis was placed at the level of resident reaction and learning that occurred immediately after completion of the intervention. Only one study repeated evaluation at the end of residency and two years post-graduation^17^ to assess for sustainable retention of attitudes, behaviours and knowledge. There were a few studies that assessed change in resident behaviour related to frequency of SDOH screening, use of resources, and referrals made to supporting services. However, this assessment was immediately post-intervention and did not look at sustainability of behaviour change. Only three studies evaluated outcomes related to academic benchmarks. These studies showed success in achieving target objectives such as scholarly work, and post-graduate engagement in leadership, advocacy, and careers focused on vulnerable populations. However, whether these curricula enhanced patient experience is unknown. Patient-related outcomes were infrequently evaluated, and reported positive results only once.^12^ One study sought to evaluate patient outcomes,^14^ but authors didn’t describe what these outcomes entailed, nor were these outcomes reported in the publication.

Although studies’ interventions and outcomes were heterogeneous, there were several recurring themes in successful curricula that had positive academic and participant related outcomes. Curriculum recommendations were extrapolated and generated from these observations. The format of a SDOH curriculum should be organized as a longitudinal experience to enable residents to have repeated exposure to elements within the program. This will solidify understanding, competence, and comfort level required to effectively manage and advocate for patients suffering from health inequities.

The content included within curriculum should vary depending on the needs of the community one serves, and should use multiple sources such as resident and community needs assessment, literature review, and local expert opinion. Programs should all include at least a basic introduction that describes the SDOH and how they impact patient care and health outcomes. Other suggested content items include resources relevant to a patient’s socioeconomic status, considerations in the patient-provider interaction, leadership and health advocacy, interprofessional collaboration, and project management.

There should be multiple types of learning activities so trainees have comprehensive exposure to the curriculum content from different perspectives. Programs should include patient or community exposure for hands-on, experiential learning. This should be supplemented and reinforced with either classroom-based and/or independent learning, or for more rigorous programs, a resident research or advocacy project.

Evaluation methods should be comprehensive and should target objectives that investigate participant, patient, program and academic related outcomes. The Kirkpatrick’s model of reaction, learning, behaviour, and results^24^ can help organize this framework. This would assure study not only of short-term outcomes such as participant satisfaction and learning, but also long-term change in physician behaviour, physician engagement in vulnerable populations, scholarly achievements, and most importantly the impact on patient care outcome.

There are a number of strengths of this scoping review. Firstly, studies found were from multiple training specialties, including curricula that incorporated several training programs. This increases the generalizability of the data, increasing the likelihood that recommendations apply to a broad range of specialty programs. Secondly, this study is a current and timely review on post-graduate curricula of SDOH, an essential topic as per the Royal College of Physicians and Surgeons of Canada, and indeed a topic that has been identified as insufficiently taught. While study interventions and outcomes were heterogeneous, recommendations could be made based on the best available evidence.

This study does have an important limitation. All studies were performed in the United States. The United States is the 12^th^ wealthiest country in the world,^25^ so less wealthy countries that may suffer greater health disparities are underrepresented.

Physicians continue to lack sufficient training in social determinants of health, an area that has a major impact on patient health. This scoping review provides recommendations for a post-graduate curriculum to extinguish this gap in physician training. While recommendations are made, this review also identifies that further research is required to clarify learning objectives and the physician’s role within the domain of health disparities.

## Appendix A

**Table:** Search Strategy through MEDLINE

#	Searches	Results	Comment
1	"internship and residency"/	44221	Residency terms
2	curriculum/ or competency-based education/ or interdisciplinary studies/ or problem-based learning/	77063	Curriculum
3	"Social Determinants of Health"/ [****MeSH since 2014 – what are the other concepts associated with social determinants i.e. SES etc.****]	1691	
4	(Social adj2 Determinant* adj2 Health).ti,ab,kf.	2660	
5	health status disparities/	12686	
6	social medicine/ [***** A branch of medicine concerned with the role of socio-environmental factors in the occurrence, prevention and treatment of disease.****]	3899	
7	health services accessibility/ or health equity/	66758	
8	exp Socioeconomic Factors/	415179	
9	or/3-8	477964	Social determinants terms
10	1 and 2 and 9	262	Base clinical set 1 - residency and curriculum and social determinants
11	Students, Medical/ or education, medical/ or education, medical, undergraduate/	91352	Medical student terms
12	program development/ or program evaluation/ or (evaluation studies or validation studies).pt.	386031	Evaluation terms
13	2 and 9 and 11 and 12	100	Base clinical set 2 - medical students and curriculum and social determinants and evaluation
14	10 or 13	352	Final results 1

## Appendix B

**Table:** Study summary

Study	Participants	Intervention	Evaluation	Outcomes
Noriea, A.H. *et al*. 2017	Internal Medicine Residents (United States)	Format: Longitudinal Content: health disparities, SDH, environmental determinants of health, patient-provider interaction, language/acculturation, disparities in research, special populations, advocacy Activities: didactic sessions, experiential learning (videos, literature evaluation, critical reflection, field trips), clinical practice, resident teaching	(i)Time/cost of implementation (ii) Resident engagement in learning sessions (iii) pre/post survey for preparedness, skill & attitude in care of vulnerable patients & commitment to change	(i) 156 hours and no external funding required to implement curriculum (ii) 21% residents participated, and attended 2.1 (of 4) didactic sessions, community exploration (38%), critical reflection (69%), video viewing (88%), resident peer development (100%) (iii) Improvement reported in 15 of 20 domains
Basu, G. *et al*. 2017	Internal Medicine Residents (United States)	Format: Longitudinal instruction separated into two-week blocks Content: 1) Health equity, SDOH & health policy 2) Health service research methods 3) Social change, leadership & advocacy Activities: Small-group sessions, didactic sessions, field trips, research and advocacy skill workshops, reflective practice, assigned reading, research-based group advocacy project	(i) End of year course evaluation (ii) Scholarly product from group health advocacy project	(i) Course rating 5.2 out of 6 (ii) All scholarly products accepted at regional and national General Internal Medicine conferences
Real, F.J. *et al*. 2016	Pediatric Residents (United States)	Format: Three 30 min teaching modules Content: Local SDH-related issues (housing, food, safe-play, pharmacies, transportation) Activities: Didactic sessions, games, case-based simulation, group discussion, resource introduction	(i) Pre/post self-assessment of SDH competence (ii) frequency considering patient neighbourhood in clinical care (iii) frequency of use of resources (iv) Child caregiver surveyed if SHD topics addressed in clinic	(i) Residents report improved competence in safe play, nutrition & transportation anticipatory guidance (ii) Caregivers felt advice given was helpful (iii) SDH topics not consistently addressed in clinic encounters
Klein, M.D. *et al*. 2014	Pediatric Residents (United States)	Format: Two 90min conferences Content: SDH screening, food insecurity, public benefits, housing conditions, maternal depression, domestic violence, education Activities: Video vignettes modeling SDH screening, patient video interviews, didactic session with faculty facilitated discussion, resource flow charts	(i) Pre/post survey of competence in screening for SDH and resource knowledge (non-intervention control) (ii) Patient assessment of trust/respect for resident and number of SDH's screened (iii) Referrals for medical-legal partnerships and formula distribution program	(i) Competence and frequency of screening for SDH higher in intervention than control (ii) Parental rating of trust/respect did not change (iii) Referral for medicolegal partnerships and formula distribution increased
Fornari, A. *et al*. 2011 AND Streinick, A.H. *et al*. 2008	Family Medicine, Internal Medicine and Pediatric Residents (United States)	Format: 3-year residency program with block-style rotations once per year. Content: Behavioural science - race, ethnicity, gender, socioeconomic class & urban environment. Interview skills & doctor-patient relationship, health & mental health assessments at patient, family, community level, & intervention skills. Social medicine: Medical Spanish, evidence-based medicine, health systems. Complementary and alternative medicine. Activities: Intercollaboration with allied health professionals, shared care of patient cohort with co-resident in community health center and hospital, monthlong orientation, small-group sessions, readings, lectures & discussion, critical literature appraisal, role-playing, & debates, home visits, field trips to communities and agencies, social medicine project & presentation of proposal.	(i)Pre/posttest knowledge exams (ii)Self-assessment of confidence (iii) post-grad career tracking with quasi-experimental design using applicant controls who trained elsewhere (iv) self-reflective essay (v) written and verbal resident course evaluations (vi) Faculty survey (vii) published scholarly activity	(i)Exam scores for knowledge increased (ii) significant attitudinal and confidence changes (iii) 50 graduates with MPHs, majority practice in underserved areas, 58% in leadership position (v) Strengths reported: thematic organization, cultural activities, community visits, advocacy lunches, small-group case discussions, cross-track collaboration of residents. Reported areas for improvement: more time to reflect/debrief, travel logistics, aligning advocacy lunches with monthly themes, using patient families from residents own clinic site (vii) 17 publications, 36 presentations at national meetings
Kuo, A.K. *et al*.2010	Pediatric Residents (United States)	Format: Longitudinal Content: Leadership development, team building, negotiation, & conflict management. Health disparities, SDOH, health policy, economics, immigrant health, foster care. Skills: community partnerships, literature search, community asset mapping, project management, grant writing, program evaluation, budget development, organizational structure, developing logic models, addressing sustainability, & communicating results. Activities: Leader shadowing, advocacy health project, advisory groups with faculty and peers, small-group seminars, community agency visits, critical reflection, discussion with local experts.	(i) Resident electronic portfolio (ii) Multisource feedback and personal reflection (iii) Entrance and exit evaluations on reasons for applying to program, satisfaction with program, impact on long-term career goals, impact on plan to influence population health and policy, plans to serve underserved population, competence as a leader, impact on clinical education/skills	- Skill-based, rather than topic-based curriculum - Residents exposed to examples of career paths for physician-leaders - Emphasis changed from underserved medicine to leadership skills and development - Residents received grants to support their health project, local/national awards in leadership and commitment to community, leadership positions in medicine
Klein, M. & Vaughn, L.M. 2010	Pediatric Residents (United States)	Format: 1-day experience in collaboration with medical-legal partners at a primary care center. Content: Medical-legal partnerships, budgeting on fixed income, public benefits, housing, educational rights & laws. Activities: Didactic sessions & field trips to local benefit organization and food bank.	(i) Resident self-reflection essay on lessons learned and how knowledge will influence practice. (ii) Reflections analyzed qualitatively for themes using “constant comparison” technique	(i) Realization regarding family circumstances effect on medical outcomes. (ii) Commitment to screening socioeconomic and environmental issues. (iii) Awareness regarding self and personal practice. (iv) Knowledge acquisition about advocacy issues and community partnerships.
Gregg, J. *et al*. 2008	Internal Medicine Residents (United States)	Format: 8 days total incorporated once/week during ambulatory medicine blocks. Content: Homelessness, poverty, continuity of care, health policy, health care safety net, motivational interviewing, complementary/alternative medicine, addiction & chronic pain. Activities: Didactic sessions, clinical exposures, field trips (homeless outreach, detox center, treatment facilities) with resident tailoring experience based on interest (addiction, primary care or community outreach)	(i) Pre/post curriculum, end of residency & 2-years postgrad self-evaluation on attitude, behavior & knowledge. (ii) Anonymous reflections and taped wrapup sessions.	(i) Resident change in how they view homeless/addicted patients.
McDougle, L. 2006	Family Medicine Residents (United States)	Format: Longitudinal Family Residency program. Content: Cultural competency, ATLS, HIV/AIDs Activities: Clinical rotations in relevant specialties, urban health research project, community clinics with predominantly African-Americans who are at or below poverty line.	(i) Survey of resident and Faculty demographics (ii) Statements from program graduates (iii) Formative/summative evaluation of attitudes/knowledge/skills (iv) Community members rate of service use and patient care outcomes.	(i) More underrepresented minority Residents and Faculty recruited.
Furin, J. *et al*. 2006	Internal Medicine Residents (United States)	Format: Four-year health equity residency program. Content: Epidemiology, health policy, ethics, medical anthropology, SDOH & disease, clinical skills for care in resource poor-settings, research in health disparities/global health, advocacy, leadership & operational management of global health programs, ethics of international medical practice/research. Activities: Clinical/research mentorship, didactic seminars, graduate-level courses, research project, site visits to health equity organizations (e.g. WHO), clinics in resource poor communities.	(i) Residents assessed for achievement of specific core competencies (ii) Health disparities research project (iii) Tracking of continued post-graduate work in health disparities field (iv) Amount fund raised through donations and grants.	(i) Core Competencies: SDOH & disease, clinical skills for care in resource poor-settings, research in health disparities/global health, advocacy, leadership & operational management of global health programs, ethics of international medical practice/research.
Jacobs, E. *et al*.2003	Internal Medicine and Pediatric Residents (United States)	Format: Four half-day sessions over one month. Content: Socioeconomic status and health, cross-cultural communication, communityoriented primary care. Activities: Didactic lectures, case-based discussion, readings & assignments, community tour, facilitated discussions, patient interviews. Partnership with nonprofit community advocacy organization with teaching shared by faculty, sociologist and community teachers.	(i) Post curriculum evaluations by both residents and community teachers.	(i) Community teachers value open discussions with residents. (ii) Residents value insight into patient lives and changed perceptions of patients but found the rotation to be short with inadequate time to gain full understanding.
Eddy, J.M. & Labuguen R.H. 2002	Family Medicine Residents (United States)	Format: Longitudinal over PGY2 of residency. Content: Underserved patient populations and the uninsured poor. Activities: Weekly half-day clinic with underserved patients.	(i) Standard elective evaluation forms to assess strengths/weakness and perception of educational experience.	(i) High level of resident satisfaction with learning experience, applicability to training, and understanding aspects of care for underserved. (ii) Weaknessess: low patient volume, and lack of on-site preceptor in some clinics
